# Francis Crick's Legacy for Neuroscience: Between the α and the Ω

**DOI:** 10.1371/journal.pbio.0020419

**Published:** 2004-12-14

**Authors:** Ralph M Siegel, Edward M Callaway

## Abstract

The legacy of Francis Crick is explored by two scientists who were influenced by his work

*‘You’, your joys and your sorrows, your memories and your ambitions, your sense of personal identity and free will, are in fact no more than the behavior of a vast assembly of nerve cells…” —Crick (1994, p. 3)*

Francis Crick was an evangelical atheist. He believed that scientific understanding removed the need for religious explanations of natural phenomena. From James Watson's and his early work, the structure of DNA explained the α, the origins of life. This was a starting point; from the elucidation of the structure of DNA, there was an explosion, a massive diversity of science that in part removed the need to postulate a creator or a creation myth. Francis still felt that life was no less astonishing just because it was biological and natural in origin. He had a consistent and completely rational world view without a need to invoke vitalism, or any non-material force (M. Crick 2004). And in the last decades of his life, he applied this philosophy to the Ω, consciousness.

Once the structure of DNA was known, the physicist George Gamow formed the RNA Tie Club, with Francis and eighteen others including his close friends Leslie Orgel and Sydney Brenner (2001); it was an ingathering that sowed seeds for future molecular biologists (Judson 1996). DNA had become the “α,” the beginning (Bronowski 1978), not just of Francis's career, but of a whole new culture of scientific life and understanding (Crick 1966).

Ten years later, the secrets of DNA transcription and translation unmasked, Francis turned to consciousness. He admitted he knew little at first, only that the structure of consciousness was as tough a problem as DNA's structure. DNA was certainly not played out, but the Ferrier Lectures in the Proceedings of the Royal Society of London by David Hubel and Torsten Wiesel were just available, tempting Francis with an almost physicist's view of neurons in action. Hubel and Wiesel wrote of functional architectures, embedded in beautiful, almost crystalline structure. The comprehension of mind invoked by a biological mechanism appeared ripe for the sort of thoughtful, theoretical science he had applied to DNA. Francis was now sixty years old and moved from Cambridge to the Salk Institute in La Jolla, California. Francis began with the brightest young minds he could find.

David Marr was a young mathematician and physiologist whose doctoral thesis on a theory of mammalian brain function at Cambridge had brought him into some contact with Brenner and Francis. A professor at the Massachusetts Institute of Technology, he began working with Tomasio Poggio of the Max Plank Institute in Tübingen on a computational theory of neuroscience. Following an invitation from Francis, Poggio and Marr spent the month of April, 1979 extending their intense examination of the core problems of visual perception. They spent hours sitting at the most western end of the Salk Institute, at the cafeteria or in Francis's office, gazing into the Pacific Ocean with all its daily changes, discussing not only architecture of visual cortex and visual perception, but the ramifications of a good theory of brain function. We know of these conversations, as the probing of Marr by Francis is captured in the final chapter of Marr's now classic book “Vision” (Marr 1982). (Although Marr speaks of a three-way conversation, judging from our own experiences as Francis's younger colleagues, the interlocutor simply seems to be Francis.)

Marr had been diagnosed with acute leukemia in the winter of 1978 (Marr and Vaina 1991). The one-month visit to the Salk Institute was an intellectual gift, for eighteen months later, Marr died. Francis had simultaneously lost a young friend and colleague who had brought an “incisive mind and creative energy” (Crick 1994, p. 77) and his best new ideas of a theoretical neurology to the brain (Marr 1969, 1970). And he saw the tragedy of Marr being cut off from solving the big problems for which he was so clearly destined.

During those early years, Francis must have thought that consciousness was tractable—if only the right way of thinking was brought to bear on it. Francis's brain was capable of collecting and filing away many disparate data, which he could then combine uniquely and imaginatively, leading to that “dramatic moment of sudden enlightenment that floods the minds when the right idea clicks into place” (Crick 1990, p. 141). Whatever his initial thoughts about the nature of the problem, Francis soon came to realize that the problem of consciousness was even tougher than he imagined, that the “click” was not happening with consciousness. In 1988, he wrote, “I have yet to produce any theory that is both novel and also explains many disconnected facts in a convincing way” (Crick 1990, p. 162).

Over the quarter century he was at the Salk Institute, Francis did propose solutions to some smaller problems in neuroscience (Sejnowski 2004) and brought consciousness into the scientific fold (Rich and Stevens 2004). But something else was going on quietly and behind the scenes. Francis was building an army to help him take on consciousness. This was not empire building with Francis as the head of a group of directed scientists in the Cambridge or German model. Francis continually encouraged and assisted young scientists to approach the hardest problems of the brain. Marr and Poggio were just the first recruits he helped embolden. He started his long-time collaboration with Christof Koch, once a post-doctoral trainee with Poggio, on “The Problem of Consciousness” (Crick and Koch 1990, 1992). His door was always open to graduate students, postdoctoral trainees, faculty who wanted to discuss those problems as many others and we can attest. Francis could be found daily at tea time, an ingathering of the Salk Institute computational and vision laboratories of Simon LeVay, Terry Sejnowski and Thomas Albright, surrounded by graduate students and post-doctoral trainees, with conversation ranging across science—Francis listening to their stories of their explorations and encouraging them to reach beyond their horizons. Francis had a “love of the truth and helped others to move to the truth” (Watson 2004).[Fig pbio-0020419-g001]

**Figure pbio-0020419-g001:**
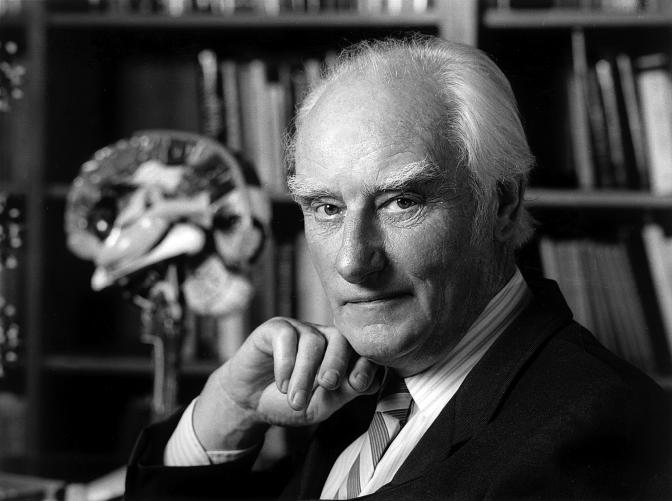
Francis Crick in his office. Behind him is a model of the human brain that he inherited from Jacob Bronowski. (Photo: Marc Lieberman)

When Francis worked on the structure of DNA, he had some simple facts, such as Chargaff's Laws, and means to make point mutations from which it could be determined how function followed structure. But not a single neuroanatomist knew how many neurons actually converged in their input to a particular single cell. No one knew how to eliminate a specific cell type from a circuit— to make a point mutation, so to speak, in the structure of consciousness. His 1979 article in Scientific American, “Thinking about the Brain,” did not have much impact at the time, even when it explicitly described three needed methods: first, a method by which all the connections to a single neuron could be stained; second, a method by which “all neurons of just one type could be inactivated, leaving the others more or less unaltered”; and third, a means to differentially stain each cortical area, “…so that we could see exactly how many there are, how big each one is and exactly how it is connected to other areas.” By the mid-1980s, Francis had realized that these massive holes in our understanding of the most simple brain facts were not being filled. Something needed to be done.

Over the twenty years since the RNA Tie Club, molecular biology had matured. Francis actively began encouraging the inclusion of the critical tools of molecular biology in the study of neural circuits and perception; in his thinking, molecular biology was critical to understand how the brain worked because it provided tools. He would encourage junior scientists, postdoctoral trainees, and faculty—all those who had visited him over the years—to think about using these tools. He would give short homilies about the plethora of sub-types of neurons in the retina; would not the cortex be at least as rich in possibilities? Molecular tools could unravel this knot.

As we reminisced after Francis's death, we discovered that Francis had spoken with each of us on these molecular methods, across a twenty-year interval. In the mid-1980s, Francis spoke with Ralph, pressing him to consider how he might do highly specific lesions of single neuron types in motion cortex using molecular identifiers. At the time, the only tools imaginable were some sort of killer antibody approach. Twenty years later, Ed recalls Francis continuing to encourage this cross-disciplinary molecular and systems approach. It was absolutely imperative to Francis's vision of the maturation of neuroscience that there would be a conjoining of molecular biology and systems neuroscience. We are sure we were not unique in hearing this call; with how many others had he shared his vision?

The science of the mind is a thinker's game. It is chess against the grandest masters, biological evolution and natural selection—and we are just learning to move the pieces. Our viewpoint is often myopic, with our noses pressed against the back row of the chessboard. It is hard to see the pieces, let alone their arrangement or the strategies they are forming. Francis may not have had the overview needed to reveal evolution's gambit, but he knew the moves needed to clear the “tangle of difficulties” (Crick 1994, p. 77) that prevented an unfogged view of his opponent's pieces.

Francis hoped for simplicity. He wrote, “Curiously enough, in biology it is sometimes those basic problems that look impossibly difficult to solve which yield most easily…. The biological problems that are really difficult to unscramble are those where there is almost infinity of plausible answers and one has to painstakingly attempt to distinguish between them.” (Crick 1990, p. 157–158). Watson and Crick had picked the right pieces of information to construct their model. Francis early on had had the same hopes to open the doors of consciousness (to paraphrase Huxley 1963). Watson and Crick used their intuition to fill in the gaps. But Francis found that there were just too many possibilities, and the gaps in knowledge were still just too big for consciousness.

In 1999, Francis felt that gentle and informal direction was not enough. Thus, he convened a meeting of molecular biologists and neuroscientists at the Salk Institute to encourage them to work together. He brought scientists including Tom Albright, Ursula Bellugi, Ed Callaway, Rusty Gage, Steve Heinemann, Terry Sejnowski, Chuck Stevens, and Inder Verma into one room and said it was time to get serious. He reminded them of the advantages of genetic methods for targeting specific cell types within complex neural circuits, and he reiterated the need for methods that could be used to identify, manipulate, and observe neural circuits in action. Not only were methods to be used in transgenic mice, but also methods based on viral vectors were needed to study the visual system of monkeys. From this, a number of initiatives moved forward, with studies ranging from the molecular biology of Williams syndrome to basic molecular tool building (Naldini et al. 1996; Blomer et al. 1997; Bellugi et al. 1999; Pfeifer et al. 2001; Zhao et al. 2001; Kaspar et al. 2002a, 2002b, 2003; Lechner et al. 2002; Lein et al. 2004).

Today the tools are emerging at an ever faster pace, at least in part due to Francis's maneuvers behind the scenes and his encouragement of junior scientists. Time is curing Francis's bout of scientific prematurity (Stent 1972). Individual cell types will soon be reversibly inactivated (Johns et al. 1999; Lechner et al. 2002; Slimko et al. 2002; Ibanez-Tallon et al. 2004); viral methods of tracing connections will start to fill in the gaps; new sensor methods for simultaneously recording from hundreds and thousands of identified neurons are coming (Guerrero and Isacoff 2001; Zemelman and Miesenbock 2001; Tsien 2003). There is a new field Francis termed “molecular psychology” or “molecular biology of systems neuroscience”; Albright simply calls it Neuroscience.

In 2001, Francis was diagnosed with colon cancer. He realized that the problem of the neural correlates of consciousness might outlast him. Francis was walking with a cane, still not waiting for anyone, nor allowing anyone to wait for him. He continued to find time for new faces in the field and continued to work on consciousness. While he had made many strides forward, he saw the race for him was winding down. He had had his hope for understanding the structure of consciousness. He had laid the groundwork. He decided to encapsulate his ideas in a “Framework” paper with Koch (Crick and Koch 2003). For many of us it was clear that he was laying out where he would go, had he enough time.

Each of the points of the Framework could form a major research initiative. Perhaps they should. But the central point is the *approach* to understanding consciousness; it is both structural and functional, peering forward into the future into what the shape might be. It was clear to his friends and colleagues that Francis was leaving a last testament.

As the cancer finally caught up with Francis, he focused on the role of the rarely studied claustrum (Sherk 1986). He wrote internal memos, brought friends and colleagues to working lunches at home with Odile, his wife of fifty-five years. Why do this? Why all this focus on another part of the brain, when only months remained? (Indeed it turned out to be weeks.) Was it his way of saying goodbye, of bringing his extended family close again? We think not. Francis wanted to make sure his plan went forward. He stressed to his visitors queries about the origins of the claustrum, its molecular biology, its role in consciousness. He was using his framework, pointing out the route to understanding the Ω of his career.

Francis was doing what he truly loved to his last moments. He needed to be doing science, perhaps more than ever, to take him away from the physical pain that he surely felt. He had built his army. Perhaps none of us even knew we had enlisted, but we had. And he was setting us off on the long march forward into a time that soon would not be for him. Francis died on the cusp of a new age of molecular systems neuroscience. Soon, we will have the tools and the data, but we will not have Francis. Francis had existed between the α of DNA and the Ω of consciousness. And for a man who never believed in the afterlife, he had indeed achieved immortality.

## References

[pbio-0020419-Bellugi1] Bellugi U, Lichtenberger L, Mills D, Galaburda A, Korenberg JR (1999). Bridging cognition, the brain and molecular genetics: Evidence from Williams syndrome. Trends Neurosci.

[pbio-0020419-Blomer1] Blomer U, Naldini L, Kafri T, Trono D, Verma IM (1997). Highly efficient and sustained gene transfer in adult neurons with a lentivirus vector. J Virol.

[pbio-0020419-Brenner1] Brenner S (2001). My life in science.

[pbio-0020419-Bronowski1] Bronowski J (1978). Magic, science, and civilization.

[pbio-0020419-Crick1] Crick FH (1979). Thinking about the brain. Sci Am.

[pbio-0020419-Crick2] Crick F (1966). Of molecules and men.

[pbio-0020419-Crick3] Crick FH (1990). What mad pursuit: A personal view of scientific discovery, reprint ed.

[pbio-0020419-Crick4] Crick FH (1994). The astonishing hypothesis: The scientific search for the soul.

[pbio-0020419-Crick5] Crick F, Koch C (1990). Some reflections on visual awareness. Cold Spring Harb Symp Quant Biol.

[pbio-0020419-Crick6] Crick F, Koch C (1992). The problem of consciousness. Sci Am.

[pbio-0020419-Crick7] Crick F, Koch C (2003). A framework for consciousness. Nat Neurosci.

[pbio-0020419-Crick8] Crick M (2004). Remembering Francis Crick—A celebration [presentation].

[pbio-0020419-Guerrero1] Guerrero G, Isacoff EY (2001). Genetically encoded optical sensors of neuronal activity and cellular function. Curr Opin Neurobiol.

[pbio-0020419-Huxley1] Huxley A (1963). The doors of perception.

[pbio-0020419-Ibanez-Tallon1] Ibanez-Tallon I, Wen H, Miwa JM, Xing J, Tekinay AB (2004). Tethering naturally occurring peptide toxins for cell-autonomous modulation of ion channels and receptors in vivo. Neuron.

[pbio-0020419-Johns1] Johns DC, Marx R, Mains RE, O'Rourke B, Marban E (1999). Inducible genetic suppression of neuronal excitability. J Neurosci.

[pbio-0020419-Judson1] Judson HF (1996). The eighth day of creation: Makers of the revolution in biology, expanded ed.

[pbio-0020419-Kaspar1] Kaspar BK, Erickson D, Schaffer D, Hinh L, Gage FH (2002a). Targeted retrograde gene delivery for neuronal protection. Mol Ther.

[pbio-0020419-Kaspar2] Kaspar BK, Vissel B, Bengoechea T, Crone S, Randolph-Moore L (2002b). Adeno-associated virus effectively mediates conditional gene modification in the brain. Proc Natl Acad Sci U S A.

[pbio-0020419-Kaspar3] Kaspar BK, Llado J, Sherkat N, Rothstein JD, Gage FH (2003). Retrograde viral delivery of IGF-1 prolongs survival in a mouse ALS model. Science.

[pbio-0020419-Lechner1] Lechner HA, Lein ES, Callaway EM (2002). A genetic method for selective and quickly reversible silencing of mammalian neurons. J Neurosci.

[pbio-0020419-Lein1] Lein ES, Zhao X, Gage FH (2004). Defining a molecular atlas of the hippocampus using DNA microarrays and high-throughput in situ hybridization. J Neurosci.

[pbio-0020419-Marr1] Marr D (1969). A theory of cerebellar cortex. J Physiol.

[pbio-0020419-Marr2] Marr D (1970). A theory for cerebral neocortex. Proc R Soc Lond B Biol Sci.

[pbio-0020419-Marr3] Marr D (1982). Vision: Computational investigation into human representation and processing of visual information.

[pbio-0020419-Marr4] Marr D, Vaina LM (1991). From the retina to the neocortex: Selected papers of David Marr.

[pbio-0020419-Naldini1] Naldini L, Blomer U, Gage FH, Trono D, Verma IM (1996). Efficient transfer, integration, and sustained long-term expression of the transgene in adult rat brains injected with a lentiviral vector. Proc Natl Acad Sci U S A.

[pbio-0020419-Pfeifer1] Pfeifer A, Brandon EP, Kootstra N, Gage FH, Verma IM (2001). Delivery of the Cre recombinase by a self-deleting lentiviral vector: Efficient gene targeting in vivo. Proc Natl Acad Sci U S A.

[pbio-0020419-Rich1] Rich A, Stevens CF (2004). Obituary: Francis Crick (1916–2004). Nature.

[pbio-0020419-Sejnowski1] Sejnowski TJ (2004). In memoriam: Francis H.C. Crick. Neuron.

[pbio-0020419-Sherk1] Sherk H, Jones EG, Peters A (1986). The claustrum and the cerebral cortex. Cerebral cortex.

[pbio-0020419-Slimko1] Slimko EM, McKinney S, Anderson DJ, Davidson N, Lester HA (2002). Selective electrical silencing of mammalian neurons in vitro by the use of invertebrate ligand-gated chloride channels. J Neurosci.

[pbio-0020419-Stent1] Stent GS (1972). Prematurity and uniqueness in scientific discovery. Sci Am.

[pbio-0020419-Tsien1] Tsien RY (2003). Imagining imaging's future. Nat Rev Mol Cell Biol.

[pbio-0020419-Watson1] Watson J (2004). (2004) Remembering Francis Crick—A celebration [presentation].

[pbio-0020419-Zemelman1] Zemelman BV, Miesenbock G (2001). Genetic schemes and schemata in neurophysiology. Curr Opin Neurobiol.

[pbio-0020419-Zhao1] Zhao X, Lein ES, He A, Smith SC, Aston C (2001). Transcriptional profiling reveals strict boundaries between hippocampal subregions. J Comp Neurol.

